# Shenfu Injection attenuates rat myocardial hypertrophy by up-regulating miR-19a-3p expression

**DOI:** 10.1038/s41598-018-23137-4

**Published:** 2018-03-16

**Authors:** Zhu-Jun Mao, Quan-Long Zhang, Jia Shang, Ting Gao, Wen-Jun Yuan, Lu-Ping Qin

**Affiliations:** 1 Department of Pharmacognosy, Zhejiang Chinese Medical University School of Pharmacy, Hangzhou, 310053 ZJ China; 2 Department of Physiology, Ningxia Medical University, Yinchuan, 750004 NX China; 3 Department of Physiology, Second Military Medical University, Shanghai, 200433 China

## Abstract

Shenfu Injection (SFI) is a classical Chinese medicine used to treat heart failure. Our previous study demonstrated that miRNAs underwent changes in rats with myocardial hypertrophy induced by abdominal aortic constriction. Interestingly, there was a significant change in miR-19a-3p, whose target gene is known to be associated with MEF2 signaling. However, whether and how SFI regulates miR-19a-3p in the treatment of myocardial hypertrophy has not been investigated. The purpose of the present study was to investigate the regulatory effect of SFI on miR-19a-3p in MEF2 signaling in the rat hypertrophic myocardium. We found that the miR-19a-3p expression level was significantly decreased in the hypertrophic myocardium, and MEF2A was the target gene of miR-19a-3p. The protein expressions of MEF2A, β-MHC, BNP and TRPC1 were significantly increased in hypertrophic cardiomyocytes. MiR-19a-3p was up-regulated after SFI treatment, and the protein expressions of these genes were significantly decreased. In addition, miR-19a-3p over-expression in hypertrophic cardiomyocytes could decrease MEF2A mRNA and protein expressions, and anti-miR-19a-3p showed the opposite result. Our study provided substantial evidence that miR-19a-3p played a functional role in MEF2 signaling in myocardial hypertrophy. SFI attenuated cardiomyocyte hypertrophy probably through up-regulating or maintaining the miR-19a-3p levels and regulating the MEF2 signaling pathway.

## Introduction

Cardiac hypertrophy is a phenomenon in which cardiomyocytes transform from a mature “contractile state” to an “embryonic synthesis state” as a compensatory response of the myocardium to various stimuli. The main pathological change is an increase in the myocardial cell volume and myocardial cell protein synthesis^1^. Left ventricular hypertrophy (LVH) is an independent risk factor for the development of clinical events such as heart failure (HF), cardiac arrhythmias, stroke and cardiovascular mortality.

Natural medicines perform well in clinical practice and have a bright future in the treatment of HF. Shenfu injection (SFI) is a traditional Chinese formula widely used for the clinical treatment of cardiovascular diseases including HF. The active ingredients of SFI include ginsenoside and aconitine extracted from red ginseng and Radix Aconiti Lateralis Preparata using modern technologies^2^. Many clinical reports and studies have shown that SFI is convenient for emergency use by virtue of its rapid effects in tonifying the heart, improving the heart function, protecting vascular endothelial cells, regulating the immune function, attenuating inflammation and improving the ability of tolerance to hypoxia^3,4^. However, few studies have reported the mechanism of SFI at RNA transcriptional level.

MicroRNAs (miRNAs) are a novel family of highly conserved, short non-coding single stranded RNA molecules that regulate the expression of their target genes in many tissues and cells^5^. Recently, miRNAs have been studied as the diagnostic biomarkers of several chronic diseases such as diabetes, cancer and cardiovascular diseases^5^. Our previous studies found that miR-378 inhibited caspase-3 expression and attenuated ischemic injury in cardiomyocytes^6^, and that miR-199a might be a potential therapeutic target for cardiac hypertrophy or heart failure^7^. However, whether and how SFI regulates miRNA in the treatment of myocardial hypertrophy remains unclear. The aim of the present study was to see whether SFI could induce miRNA change in the rat hypertrophic myocardium and how this change affects the downstream genes.

## Results

### Effects of SFI on the rat LVH

**Figure 1:**
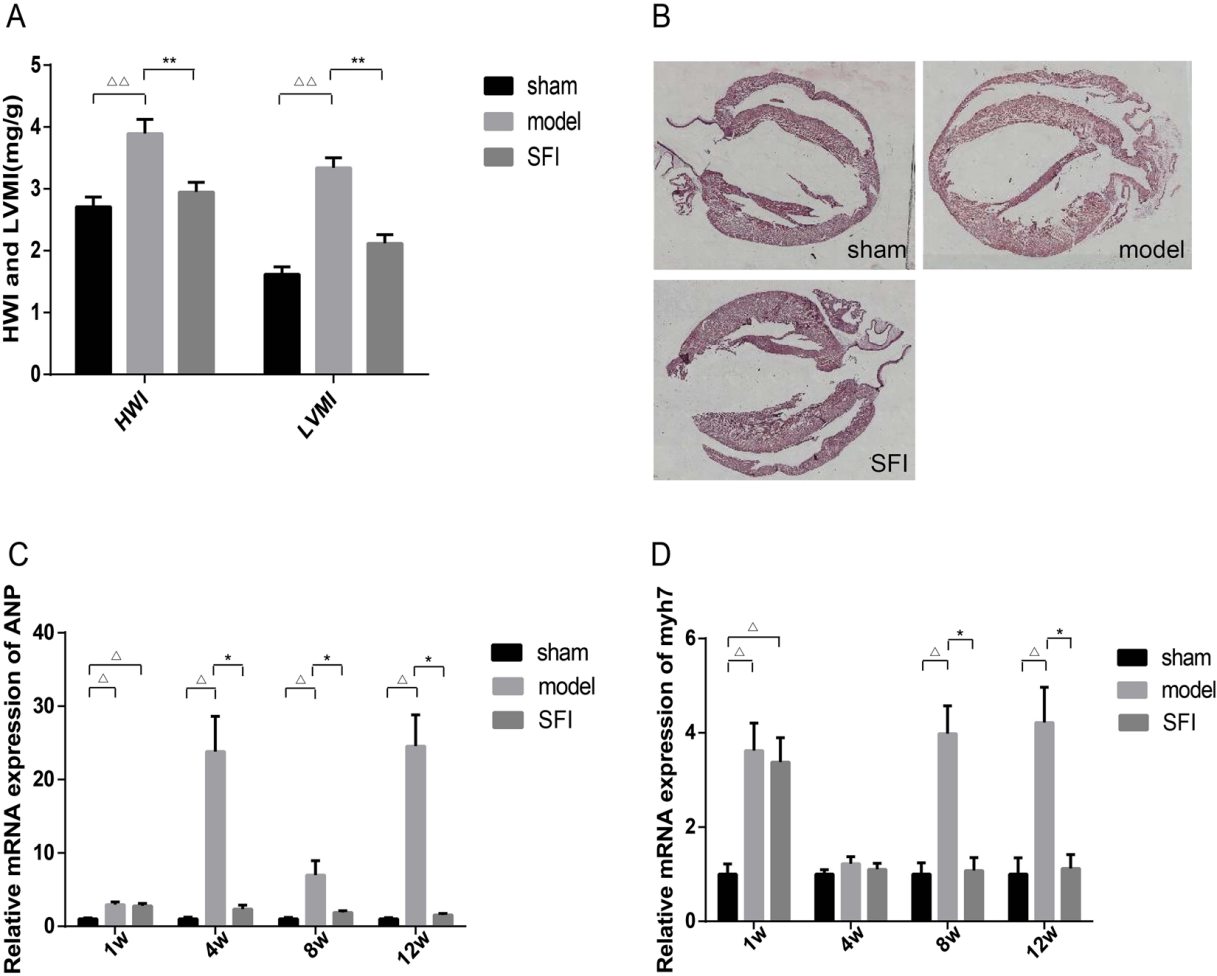
Effect of SFI on left ventricular hypertrophy-induced changes in the rat heart. (**A**) Degree of HWI and LVMI, n = 8, ^△△^p < 0.01 *vs*. sham-operation group, **p < 0.01 *vs*. model. (**B**) Effect of SFI on left ventricular hypertrophy-induced histopathological changes in the rat heart. (**C**) H&E staining of the heart specimens (n = 5), ×400). (**C** and **D**) Expression of ANP myh7 mRNA by RT-PCR. GAPDH was used as internal control. n = 8, ^△^p < 0.05 *vs*. sham-operation group, *p < 0.05 *vs*. model.

HWI and LVMI are important indexes for determining LVH or left ventricular remodeling. As shown in Fig. 1A, HWI and LVMI were elevated significantly in model group rats at 12 weeks as compared with those in sham-operation group. On the contrary, there was no significant difference between SFI group and sham-operation group.

HE staining showed that rats in model group developed LVH at 12 weeks, while no LVH was detected in SFI group at 12 weeks (Fig. 1B).

ANP and myh7 are the markers of myocardial hypertrophy. To further examine changes in myocardial hypertrophy after SFI treatment, mRNA expressions of ANP and myh7 in the rat myocardium were studied at 1, 4, 8 and 12 weeks. As shown in Fig. 1C and D, the expressions of ANP and myh7 mRNA were both increased significantly in model group as compared with those in sham-operation group at 12 weeks (p < 0.05). It was found that the expressions of ANP and myh7 mRNA were both decreased significantly in SFI group as compared with those in model group (p < 0.05), and there was no significant difference between SFI group and sham-operation group at 12 weeks.

### Differentially expressed miRNAs in the rat myocardium of the three groups

**Figure 2:**
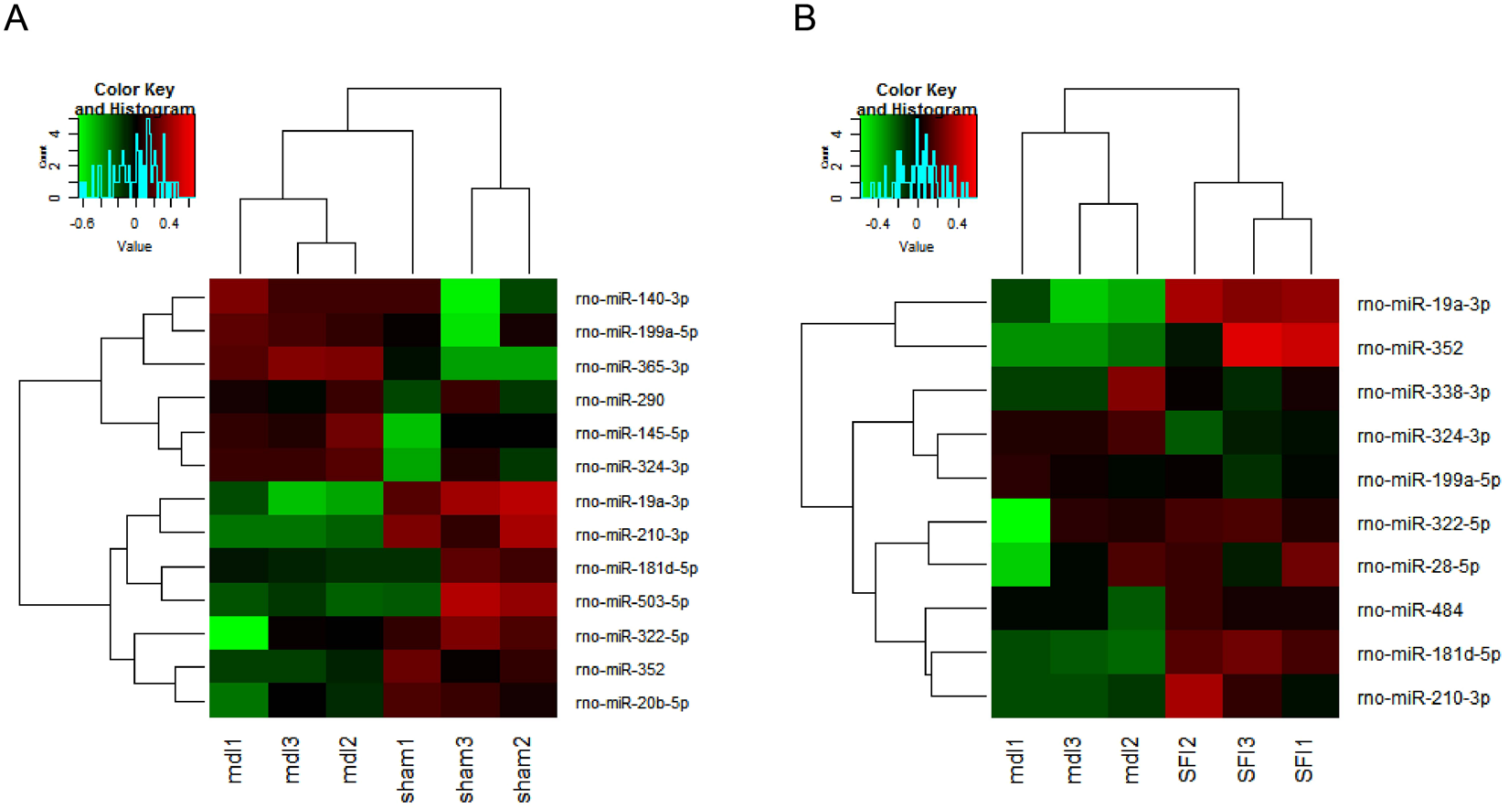
Heat map of differentially expressed miRNAs. (**A**) 13 differentially expressed miRNAs from the myocardial tissues between sham-operation group (n = 3) and model group (n = 3). (**B**) 10 differentially expressed miRNAs from the myocardial tissues between model group (n = 3) and SFI group (n = 3). Each row represents one miRNA, and each column represents a myocardial tissue sample. The legend on the right indicates miRNA represented in the corresponding row. The relative miRNA expression is depicted according to the color scale. Red indicates up-regulation, and green indicates down-regulation.

Myocardium miRNA expression was examined in model group to explore the contribution of miRNAs to myocardial hypertrophy. Altogether 13 differentially expressed miRNAs were isolated from the myocardium of model group and compared with those in sham-operation group (Fig. 2A). Altogether 10 differentially expressed miRNAs were isolated from the myocardium of SFI group and compared with those in model group (Fig. 2B).

### MEF2A is a target gene of miR-19a-3p

**Figure 3:**
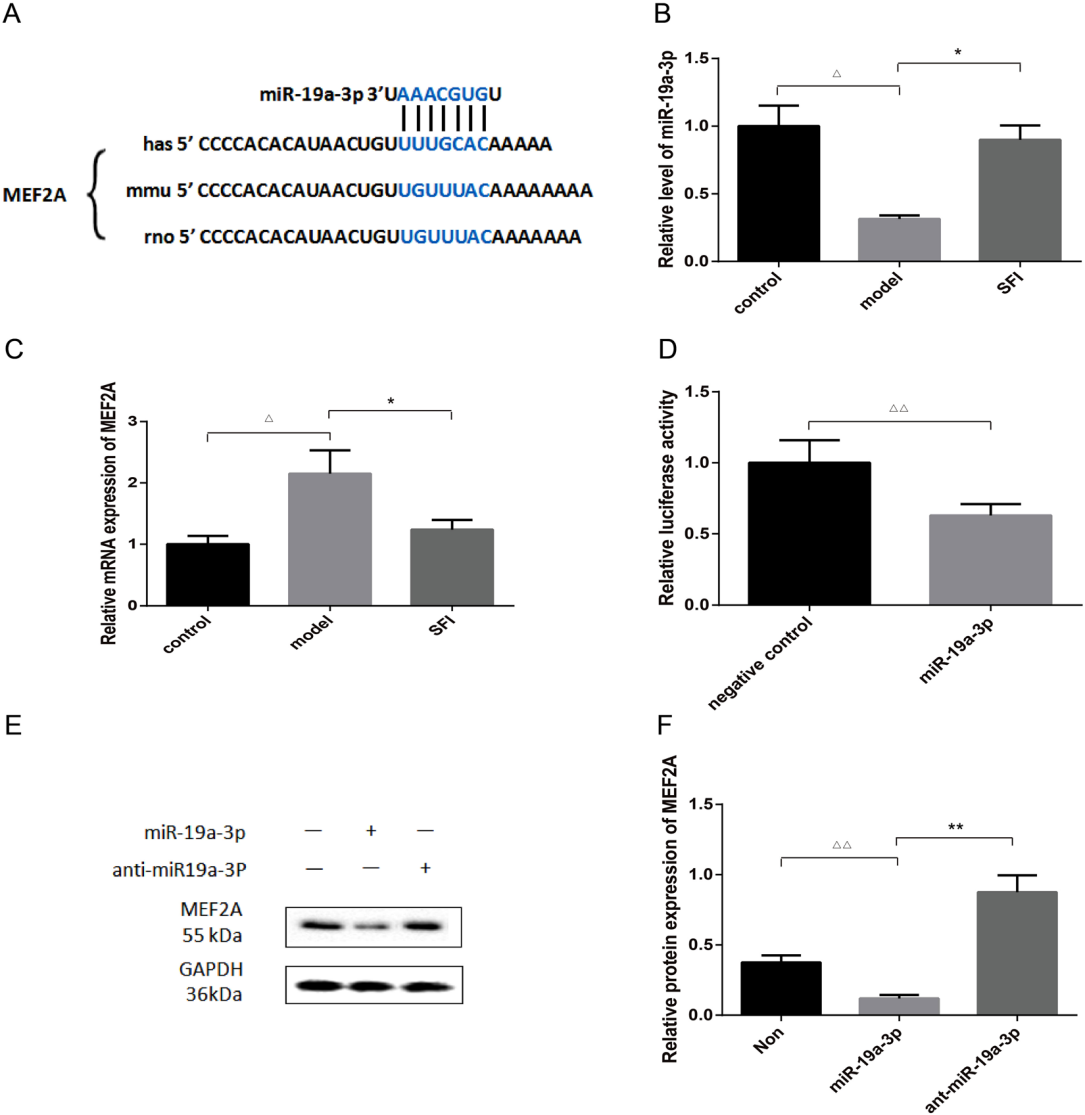
MEF2A is the target gene of miR-19a-3p. (**A**) The predicted binding site of miR-19a-3p in 3′-UTR of MEF2A mRNA. (**B** and **C**) Expressions of miR-19a-3p and MEF2A mRNA by RT-PCR, using U6 and GAPDH as the internal control, respectively. n = 8, ^△^p < 0.05 *vs*. sham-operation group, ^*^p < 0.05 *vs*. model. (**D**) Detection of the interaction between miR-19a-3p and the 3′-UTR of MEF2A by dual-luciferase reporter assay. n = 8, ^△△^p < 0.01 *vs*. NC. (**E** and **F**) MiR-19a-3p inhibited the protein expression of MEF2A after infection with miR-19a-3p mimic or miR-19a-3p inhibitor (anti- miR-19a-3p) as detected by WB analysis, using GAPDH as the internal control. n = 3, ^△△^p < 0.01 *vs*. NC, ^**^p < 0.01 *vs*. miR-19a-3p mimics.

To explore the mechanism by which miR-19a-3p affected cardiomyocyte hypertrophy, we searched potential targets of miR-19a-3p using bioinformatic algorithms, such as MiRanda, Pictar and TargetScan. Myocytes specific enhancer factor 2A (MEF2A), a critical executioner of cardiac hypertrophy, was predicted as a putative target of miR-19a-3p. The identification of one miR-19a-3p-binding site was done in the 3′ -UTR of MEF2A mRNA (Fig. 3A).

miR-19a-3p and MEF2A mRNA levels were determined. As shown in Fig. 3B and C, miR-19a-3p levels were decreased by 0.69 fold in model group after abdominal aortic constriction (AAC). Interestingly, the increase in miR-19a-3p expression was most pronounced in SFI group, by 0.58 fold (Fig. 3B). We further examined the expression of MEF2A, one confirmed target of miR-19a-3p, along with mRNA levels by qRT-PCR. It was found that the expression of MEF2A mRNA was increased significantly in model group as compared with that in control group (p < 0.05).On the contrary, there was no significant difference between SFI and control groups (Fig. 3C).

To affirm the interrelation between miR-19a-3p and MEF2A, the dual-luciferase reporter assay was implemented. Compared with the negative control RNA, the miR-19a-3p mimic significantly suppressed the activity of the luciferase reporter fused with MEF2A 3′-UTR by 37% (Fig. 3D), suggesting that miR-19a-3p inhibited MEF2A expression through its 3′-UTR.

The Western blot result showed that MEF2A was down-regulated, while anti-miR-19a-3p promoted MEF2A expression (Fig. 3E,F). Overall, these results revealed that miR-19a-3p could directly target and regulate the expression of MEF2A.

### SFI decreases the area of hypertrophic cardiomyocyte surface

**Figure 4:**
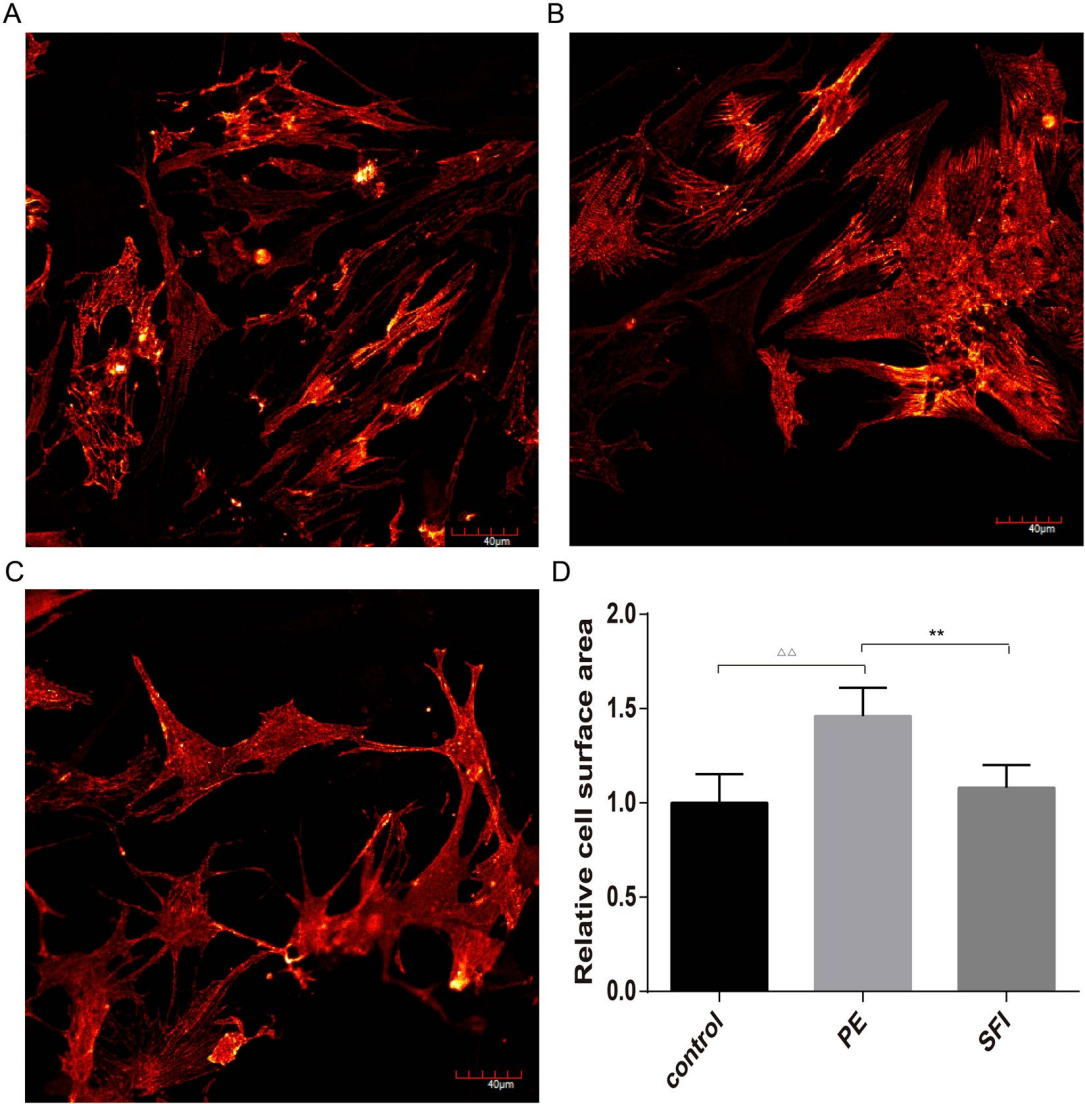
(**A**,**B** and **C**), α-actinin staining showed hypertrophy of neonatal cardiomyocytes treated with 100 M phenylephrine (PE) (SFI group treated with PE and SFI) for 48 h. Representative confocal images are shown. Scale bar: 40 μm. (**D**) quantitative analysis of cardiomyocyte size. Approximately 200 cells immunostained with anti-α-actinin antibody were randomly chosen from each treatment for surface area measurement. n = 6, ^△△^p < 0.01 *vs*. control, **p < 0.01 *vs*. model.

Immunofluorescence staining showed that the cardiomyocytes developed hypertrophy after 48-h treatment, as evidenced by the increased cell surface area (Fig. 4A,B). SFI was able to decrease the area of cardiomyocyte surface (Fig. 4C).

### SFI reduces cell size, increases the expression level of miR-19a-3p and MEF2A mRNA after transfection with miR-19a-3p mimic or inhibitor

**Figure 5:**
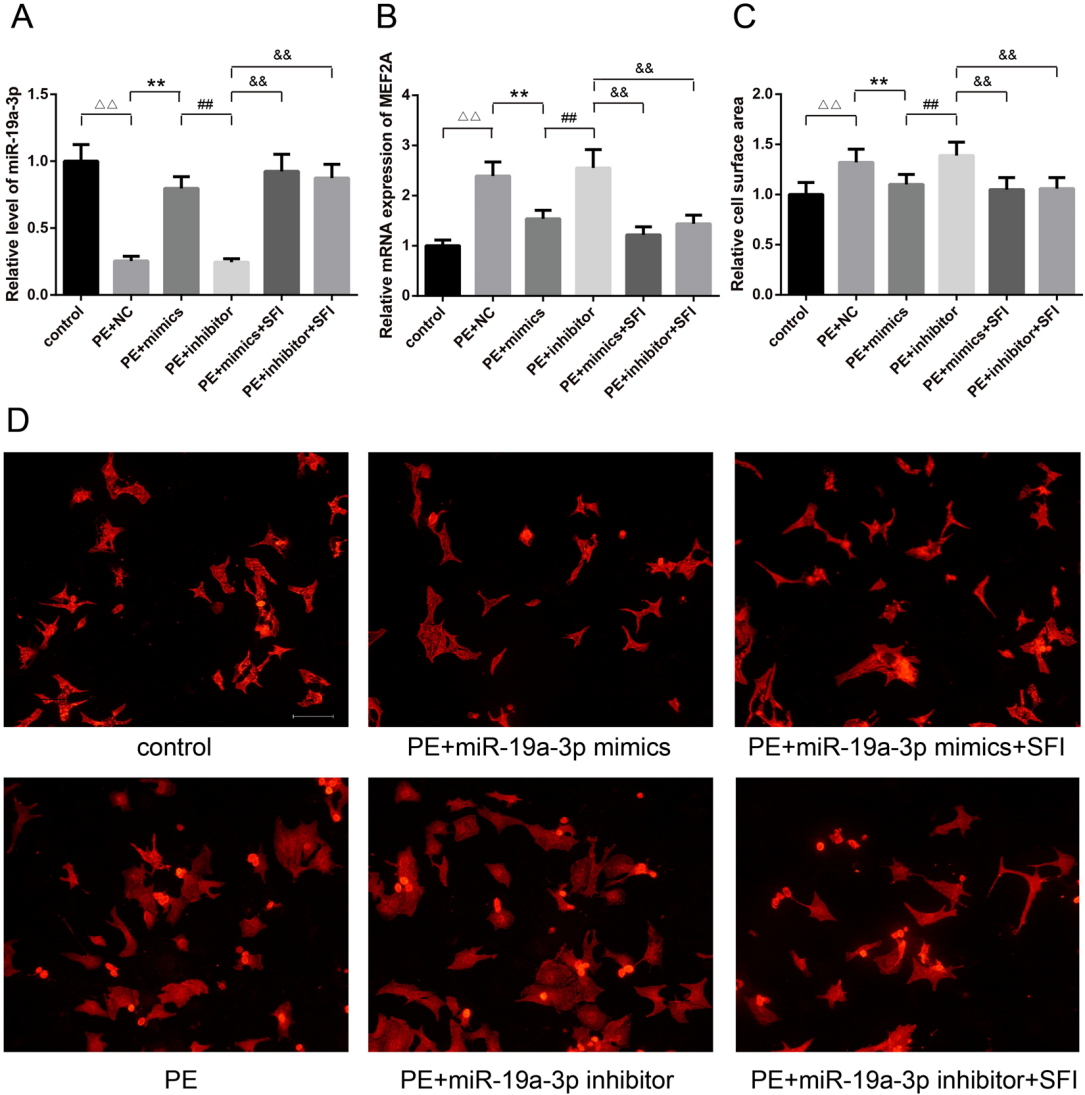
(**A**) Real-time quantitative PCR demonstrated that transfection of cardiomyocytes with miR-19a-3p mimics increased the expression of miR-19a-3p level, while miR-19a-3p inhibitor reduced the expression of miR-19a-3p level, but SFI increased the expression of miR-19a-3p level (**P < 0.01, n = 3 for each group). ^△△^p < 0.01 *vs*. control, ^**^p < 0.01 *vs*.PE + NC. ^##^p < 0.01 *vs*. PE+ miR-19a-3p mimics. ^&&^p < 0.01 *vs*. PE+ miR-19a-3p inhibitor. (**B**) MEF2A mRNA level was significantly down-regulated in miR-19a-3p mimic group, and significantly up-regulated in miR-19a-3p inhibitor group, but SFI increased the expression of MEF2A mRNA level (**P < 0.01, n = 3 for each group). ^△△^p < 0.01 *vs*. control, ^**^p < 0.01 *vs*.PE + NC. ^##^ p < 0.01 *vs*.PE+ miR-19a-3p mimics. ^&&^p < 0.01 *vs*. PE+ miR-19a-3p inhibitor. (**C** and **D**) In the medium supplemented with PE, over-expression of miR-19a-3p reduced the cell size as measured by cell surface area (100 mM). Lower-expression of miR-19a-3p increased the cell size (100 mM). SFI reduced the cell size either transfected with miR-19a-3p mimics or inhibitor. n = 6, ^△△^p < 0.01 *vs*. control, ^**^p < 0.01 *vs*.PE + NC. ^##^p < 0.01 *vs*. PE+ miR-19a-3p mimics. ^&&^p < 0.01 *vs*. PE+ miR-19a-3p inhibitor.

miR-19a-3p was significantly down-regulated in AAC 12-week hearts. Therefore, we further investigated the effect of miR-19a-3p on primarily cultured cardiomyocytes from neonatal rat. qRT-PCR analysis demonstrated that transfection of cardiomyocytes with miR-19a-3p mimics increased the expression of miR-19a-3p level (p < 0.01) (Fig. 5A). The results showed that the level of MEF2A mRNA was markedly down-regulated by 36% in miR-19a-3p overexpression cardiomyocytes compared with NC (p < 0.01) (Fig. 5B). When cardiomyocytes were transfected with miR-19a-3p mimic or NC for 48 h, up-regulation of miR-19a-3p reduced the cell size by 17% as measured by cardiomyocyte immunochemistry and cell surface area (p < 0.01) (Fig. 5C).

To investigate whether endogenous miR-19a-3p played a significant role in cardiomyocytes, antisense oligonucleotidemediated miRNA knockdown was performed using miR-19a-3p inhibitor to knock down the endogenous miR-19a-3p. The same amount of NC was used as a control. miR-19a-3p inhibitor was sufficient to knock down endogenous miR-19a-3p expression in cultured cardiomyocytes as analyzed by qRT-PCR (p < 0.01) (Fig. 5A). Compared with the miR-19a-3p mimic, the level of MEF2A mRNA was markedly up-regulated by 40% in miR-19a-3p lower-expression cardiomyocytes compared with miR-19a-3p mimic (p < 0.01) (Fig. 5B). The size of cardiomyocytes transfected with miR-19a-3p inhibitor increased by 26% in culture medium with PE (100 mM) (p < 0.01) (Fig. 5C).

The result of qRT-PCR analysis showed that the expression level of miR-19a-3p increased by 3.68-fold and 3.48-fold when the cardiomyocytes were treated with 10 um/ml SFI and transfected with miR-19a-3p mimic or miR-19a-3p inhibitor for 48 h respectively, as compared with NC group (p < 0.01) (Fig. 5A); the level of MEF2A mRNA was down-regulated by 49% and 40% respectively (p < 0.01) (Fig. 5B); the cell size both reduced by 20% (p < 0.01) (Fig. 5C)

### Effects of SFI on protein expression in the hypertrophic myocardium and cardiomyocytes

**Figure 6:**
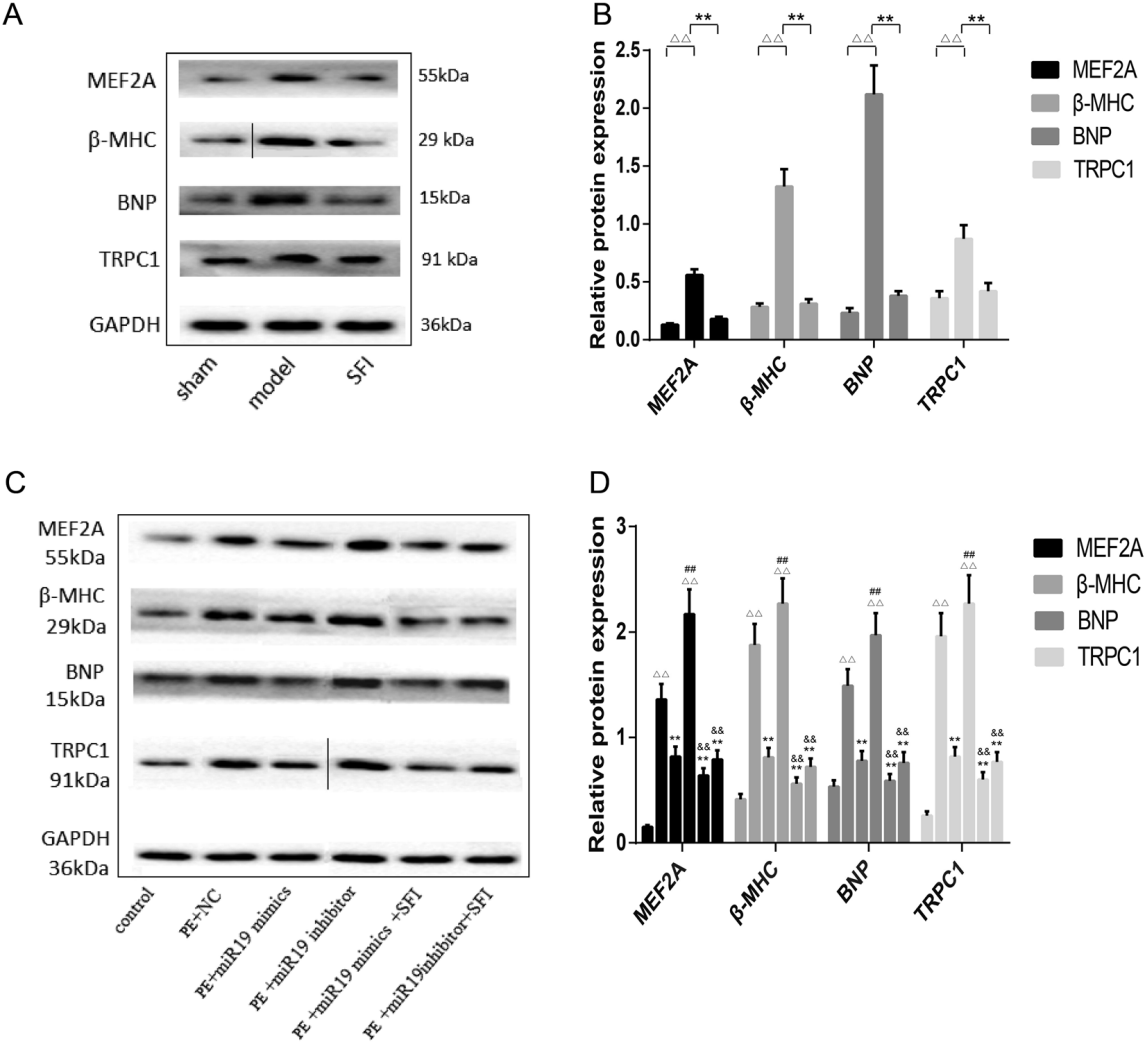
Effects of SFI on MEF2A, β-MHC, BNP and TRPC1 protein expressions. The protein expressions of MEF2A, β-MHC, BNP and TRPC1 were detected by Western blotting. (**A** and **C**) The figure represents one of three experiments with similar results. Data are presented as the means ± SD from three independent experiments. (**B**) ^ΔΔ^p < 0.01, *vs*. sham-operation group,^**^p < 0.01, *vs*. model group. (**D**) ^ΔΔ^p < 0.01, *vs*. control,^**^p < 0.01, *vs*. PE + NC, ^##^p < 0.01, *vs*. PE + miR30 mimics,^&&^p < 0.01 *vs*. PE + miR30 inhibitor.

As demonstrated in Fig. 6, in both AAC-induced hypertrophic myocardium and PE-induced hypertrophic myocyte model, the protein expressions of MEF2A, β-MHC, BNP and TRPC1 were all increased significantly as compared with those in sham-operation or control group (p < 0.01), while there was no significant difference between SFI and control groups (Fig. 6A,B). The levels of the above-mentioned proteins were decreased significantly in the clones infected with miR-19a-3p mimic in PE-induced hypertrophic myocytes (p < 0.01), among which the protein expression of MEF2A, β-MHC, BNP and TRPC1 all increased significantly in the clones infected with miR-19a-3p inhibitor (p < 0.01). After SFI treatment, MEF2A, β-MHC, BNP and TRPC1 protein expressions were decreased significantly as compared with those in model group (p < 0.01) (Fig. 6C,D).

## Discussion

The physiopathological mechanisms implicated in myocardial hypertrophy are multi-factorial, including hypertensive LVH, aortic stenosis, hypertrophic obstructive cardiomyopathy and pulmonary hypertension. Cardiac hypertrophy is mainly related to some stimulating-factor pathways, such as MAPK signaling pathway, JAK-STAT pathway, and SMAD pathway^8,9^.

MiRNAs, as important regulatory factors in the field of cardiovascular disease, have attracted increasing attention. More studies have shown that different miRNAs play important roles in cardiac hypertrophy. MiR-208a, which is encoded by MHC genes, has been shown to form an intricate regulatory circuit together with their host genes to regulate cardiac hypertrophy^10^. MiR-1 and miR-133 were down-regulated in exercised trained rats and cardiac-specific Akt transgenic mice^11,12^. In our previous study, we showed that miR-199a was dynamically regulated during hypertrophy. Initially, miR-199a increased after AAC for a week, then returned to normal levels at 4 weeks, and increased again by several fold after 12 weeks. We think that the different expression levels of miR-199a at different time points could be due to the different physiological or pathological conditions of the heart after AAC. Rats subjected to AAC for a week experienced a short-period acute reaction along with sympathetic nervous excitation and renin-angiotensin system activation^7^.

The current study aimed to investigate changes in miRNA expression in the rat myocardial hypertrophy model to assess their underlying devotion to disease development and progression, in an attempt to explore the mechanism underlying the regulation of Shenfu Injection on miRNA expression in the rat hypertrophic myocardium. Our findings indicate that particular alterations in myocardial miRNA expression existing in myocardial hypertrophy conditions reflect a pathological state of the myocardium. We found that miR-19a-3p expression underwent a change after myocardial hypertrophy. Analysis of the functional impact of individual change on miR-19a-3p expression in the myocardium revealed that it had a beneficial effect on the maintenance of a normal physiological state.

We suppose that SFI attenuates myocardial hypertrophy by upregulating the levels of particular miRNAs, including miR-19a-3p, miR-181d-5p, miR-210-3p, miR-352 and miR-324-3p, and downregulating miR-199a-5p. Our study for the first time addressed the implication of miRNAs in the hypertrophic myocardium through systematic investigations on the efficacy of SFI in regulating miRNAs in myocardial hypertrophy. Our data demonstrate that myocardial hypertrophy induced by AAC is associated with alterations in two different groups of myocardium miRNAs, which have opposing phenotypic effects on the myocardium. These alterations in miRNA expression provide clear evidence that SFI regulates miRNAs in the hypertrophic myocardium. It was found in our study that the most striking change was the miR-19a-3p expression in model group. However, SFI significantly reversed that situation. And we identified the target gene MEF2A through bioinformatic algorithms, dual-luciferase reporter assay and RT-PCR method. Our primary research question was how myocardial miR-19a-3p played a role in the MEF2 signaling pathway.

Pathologically, myocardial hypertrophy can be triggered by hypertension, valvular dysfunction and myocardial infarction^13^. Calcium-dependent phosphatase calcineurin is a key regulator of hypertrophy. Activated calcineurin dephosphorylates the nuclear factor of activated T-cells (NFAT), which subsequently translocate to the nucleus to activate genes associated with stress, such as atrial natriuretic peptide (ANP) and brain natriuretic peptide (BNP), and genes that encode fetal isoforms of contractile proteins, such as β-myosin heavy chain (β-MHC)^14,15^. In our study, mRNA expressions belonging to ANP and myh7 in the myocardium were significantly increased under the hypertrophic condition. We found that mRNA expressions belonging to ANP and myh7 were partially restored after SFI treatment.

The myocyte enhancer factor 2 (MEF2) transcription factor family has been shown to play a crucial role in the activation of muscle-specific gene transcription in skeletal, cardiac and smooth muscle cells^16^. The products of MEF2 genes are associated with many muscle-specific genes in vertebrates, such as cardiac troponins T, C or Ca^2+^-ATPase^16^. Several SNPs identified in the human MEF2A gene were reported to be associated with hypertrophic cardiomyopathy and coronary artery disease^17–19^. MEF2A, which has been shown in Knock-out studies, is essential for energy metabolism and sarcomeric organisation in adult hearts^15^. The MEF2 family is also considered an important transcription factor in myocyte hypertrophy and is involved in mediating the hypertrophic action of glucose on cardiomyocytes^20^, but the regulatory relationship with MEF2 and miRNA is less clear. Interestingly, our study showed that MEF2A was the target gene of miR-19a-3p, and it was highly expressed in the hypertrophic myocardium model under lower-expression of miR-19a-3p.

TRP channels are a family of non-selective cation channels that function as polymodal sensors in different physiological and pathological processes^21,22^. Canonical transient receptor protein 1 (TRPC1) encoded by the TRPC1 gene is an ion channel located on the plasma membrane of numerous human and animal cell types^23^. It is a non-specific cation channel through which both sodium and calcium ions can pass. TRPC1 is thought to mediate calcium entry in response to depletion of endoplasmic calcium stores or activation of receptors coupled to the phospholipase C system^24^. Functionally, TRPC1 is involved in cardiac hypertrophy, hypertension, vascular inflammation and cancer^25,26^.

However, changes of these genes in SFI- treated hypertrophic cardiomyocytes have not been studied before. In our study, the protein expressions of MEF2A, β-MHC, BNP and TRPC1 were significantly increased after PE treatment, which proved to be a state under hypertrophic conditions. Our data suggest that MEF2A is a target of miR-19a-3p, and miR-19a-3p up-regulation could reduce the expression of MEF2A mRNA and protein. Furthermore, lower-expression of miR-19a-3p may enhance the expression of MEF2A, β-MHC, BNP and TRPC1 in cardiomyocyte hypertrophy, which seemingly implies that lower-expression of miR-19a-3p mediated MEF2 signaling might be a cause of myocardial hypertrophy. Interestingly, SFI could reverse these situations.

Generally, our study adds new clues to the understanding about the action mechanism of SFI in the treatment of myocardial hypertrophy. We suppose that dysregulation and function of miR-19a-3p in the myocardium play an important role in myocardial hypertrophy. Over-expression of miR-19a-3p in the myocardium may prove to be able to attenuate myocardial hypertrophy. MEF2A is inhibited by miR-19a-3p under physiological conditions, and during the myocardial hypertrophy process as well. These results also imply that miR-19a-3p may regulate MEF2-related genes. Our study suggested that SFI attenuated myocardial hypertrophy probably by enhancing the expression of miR-19a-3p and down-regulating the expression of MEF2A, β-MHC, BNP and TRPC1, which seemingly implies that the SFI mediated improvement within MEF2 signaling might be the cause of attenuated myocardial hypertrophy.

It is worth noting that, according to the miRNA microarray data, it seems that the benefit of SFI is due to upregulation and downregulation of several miRNAs. SFI is a traditional Chinese medicine preparation, and its role in cardiac hypertrophy seems to be multi-targeting. In our current study, the regulation of miR-19a-3p expression only represents one action of SFI’s targets. In addition, viral-mediated overexpression of miR-19a-3p in the rat hypertrophy model would help understand how relevant SFI-induced upregulation of this miRNA attenuates hypertrophy, and help optimize our *in vivo* experimental studies in further.

## Conclusion

For the first time, we discovered that SFI could attenuate myocardial hypertrophy, probably through up-regulating or maintaining the miR-19a-3p level, decreasing MEF2A mRNA and protein expressions, and regulating the protein expression of β-MHC, BNP and TRPC1 of the MEF2 signaling pathway. The research results would help better understand the efficacy and action mechanism of SFI for the treatment of myocardial hypertrophy at miRNA level.

## Methods

### Transverse abdominal aorta constriction and SFI administration

After anesthesia of the SD rat with 10% chloral hydrate, the abdomen was exposed, and the abdominal aorta was dissected suprenally and tied with a 4 nylon suture against a 24 gauge needle. After removing the needle, the abdomen was closed and the rat was raised as usual. Rats in the control group underwent a sham operation without tied. One hundred and twenty rats were assigned to three groups: SFI group using 6.0 mL·kg-1·d SFI (Ya’an 39 Pharmaceutical Co., Ltd, Ya’an, China; Lot No. 131013010), model group using 6.0 mL·kg-1·d normal saline (NS), and sham-operation group using 6.0 mL·kg-1·d NS, all via intraperitoneal injection for 12 consecutive weeks. All experimental procedures conformed to the National Institutes of Health (NIH) guidelines, and approved by the Ethics Committee of the Experimental Animal Centre of Ningxia Medical University (Approval No: SVXK(Ning)2011-0001).

### Heart weight index (HWI), left ventricular mass index (LVMI) and histopathological analysis

Rat HWI and LVMI were analyzed 12 weeks after SFI treatment using the following equations: HWI = heart weight/body weight, and LVMI = left ventricular weight/ body weight.

Pathological changes in the myocardial tissue were observed by hematoxylin-eosin (HE) staining. Five to six rats randomly selected from each of the three groups and anesthetized with pentobarbital sodium (30 mg/kg IP) 12 weeks after SFI treatment. The hearts were removed, washed with NS for 10 min, and fixed in neutral-buffered 4% formalin for 48 h. Paraffin-embedded samples were sliced to 4-μm sections and HE stained^6^. Photographs were obtained using an Olympus SZX7 stereomicroscope and inverted microscope (Olympus Co., Tokyo, Japan).

### miRNA microarray analysis

RNA was extracted from the myocardial tissues in all three groups (n = 3 each) using Trizol reagent (Invitrogen, Shanghai, China), and then analyzed by miRNA microarray (Chip ID miRRat 11.0 version, LC Sciences, Houston, TX, USA), according to the description in the literature^27^. Data were analyzed by subtracting the background, and then standardizing the signal with a LOWESS filter as a regression weighted locally. The mean of three biological replicates was used as the expression level of miRNA, and compared between the three groups by Student’s t-test.

### miRNA target prediction

miRNA targets were searched using publicly available predictions. Variables selected for best targets included the following websites: MirWalk. http://www.umm.uni-heidelberg.de/apps/zmf/mirwalk., MiRanda.http://www.microrna.org/microrna/home.do., RNAhybrid. http://bibiserv.techfak.uni-bielefeld.de/rnahybrid., and TargetScan. http://targetscan.org. http://www.mir2disease.org.

### Construction of luciferase reporter plasmid and analysis of luciferase activity

To examine whether miR-19a-3p regulated the expression of MEF2A, a predicted target of miR-19a-3p, the dual luciferase psiCheck2 reporter plasmid (Promega, Madison, WI, USA), was used to generate the reporter plasmid harboring MEF2A 3′-UTR. Briefly, a 1383-bp fragment of MEF2A 3′-UTR containing the putative miR-19a-3p binding site was amplified from rat genomic DNA by PCR, using Xho1 and Not1 primers (left Xho1 primer: ctcgagCGGAGCAGAGCCATGGGCACGTCTTCAG, right Not1 primer: gcggccgcCCTATTGCTGGATGCTTTCCAAGTCCC). The PCR product was digested with Xho1 and Not1, followed by insertion into the multiple cloning region located at the 3′-UTR of the synthetic Renilla luciferase gene within the psiCheck2 plasmid. The psiCheck2 plasmid also contained a synthetic firefly luciferase gene that served as the transfection control^6^.

293 T cells were co-transfected with the psiCheck2 vector containing MEF2A 3′-UTR and miR-19a-3p mimic using Lipofectamine 2000 (Invitrogen, Carlsbad, CA,USA), and the co-transfection with non-targeting negative control RNA was performed as control. Cells were harvested 24 h after transfection, and luciferase activity was measured with a dual luciferase reporter assay kit (Thermo Fisher Scientific Inc, USA) on a luminometer (Lumat LB9507), as we described previously^6^.

### Establishment of the hypertrophic myocyte model

Neonatal rat ventricular myocytes were isolated from 1–3-day-old SD rats. Briefly, the isolated heart was digested with 0.2% trypsin at 37 °C. After dissociation, cells were subjected to centrifugation to enrich the myocytes, followed by differential preplating to delete nonmyocytes. Cells were planted at a density of 3–5 × 10^5^ in DMEM with 20% FBS. 24 h after plating, serum was removed and cells were treated with phenylephrine at 100 μM. The modeled hypertrophic myocytes were equally randomized to two groups: a SFI-treatment group, and a non-treated model group. Animals in SFI group were treated with SFI (10 μl/ml) for 48 h, using normal cardiomyocytes as the control.

### Cardiomyocyte immunochemistry and cell surface area analysis

Cardiomyocytes were fixed in 4% paraformaldehyde for 15 min and permeabilized with 0.1% Triton X-100 in PBS, followed by blockage with 5% goat serum in PBS for 1 h at room temperature. They were then incubated with anti-actinin antibody (Sigma, St. Louis, MO) at 1:500 overnight. After washing with PBS for three times, the secondary antibody coupled with Alexa-555 (Molecular Probes, Eugene, OR) was added to the cells. After washing, the slides were mounted using fluorescent mounting medium. Images were captured using a laser scanning confocal microscope camera (Olympus FLUOVIEW FV1000 Viewer, Japan). Cell surface area was analyzed using software AxioVision 4.7.1 (Carl Zeiss)^7^.

### Quantitative reverse transcriptase-polymerase chain reaction (qRT-PCR)

Atrial natriuretic peptide (ANP), β-myosin heavy chain gene (MYH7) and myocytes specific enhancer factor 2A (MEF2A) mRNA levels and miR-19a-3p levels in the myocardial tissues and/or cardiomyocytes were determined by qRT-PCR. One gram out of overall RNA elicited by Trizol (Invitrogen, Shanghai, China) was transcribed reversely by M-MLV reverse transcriptase along with oligo-dT for mRNAs and a particular stem-loop primer as TCAGTTTTGCATAGATTTGCA (5′-3′) for miR-19a-3p. The performance of PCR with real-time quantity was acted in a Rotor-Gene 3,000 real-time detection system of DNA (Corbett Research, Sydney, Australia) by SYBR Green (Qiagen, Shanghai, China) using the primers as shown in Table 1. All samples were analyzed in duplicate, containing no-template controls. The normalized curve method was used to determine the linked expression level of the mRNAs and miR-19a-3p, which was standardized to GAPDH and U6, respectively.

**Table 1:** Primers for real-time PCR.

Gene name	Forward primer (5′-3′)	Reverse primer (5′-3′)
miR-19a-3p	CAATCCTCTCAGGCTCAGTCC	TATGCTTGTTCTCGTCTCTGTGTC
MEF2A	GCAGCAGCACCACCTAGGAC	CTGCTGCTGCTGCTGGAAG
ANP	TCGTCTTGGCCTTTTGGCT	TCCAGGTGGTCTAGCAGGTTCT
Myh7B	GCAGAAGCGCAACGCAGAGT	TGCTGCACCTTGCGGAACTTG
U6	CTCGCTTCGGCAGCACA	AACGCTTCACGAATTTGCGT
GAPDH	TGGCCTCCAAGGAGTAAGAAAC	GGCCTCTCTCTTGCTCTCAGTATC

### Cardiomyocyte transfection

Cells were cultured according to the method described above. The specific mimic or inhibitor RNA was transfected to overexpress or knock down miR-19a-3p expression. For transfection experiments, cardiomyocytes were seeded at a density of 2 × 9–10^4^ cells/cm^2^ in serum-free DMEM, with addition of the transfection agent and RNAs. Cells were divided into six groups: control group (normal cardiomyocytes), PE + NC group (PE model transfected with negative control), PE + miR19 mimics group (PE model transfected with miR-19a-3p mimics), PE + miR19 inhibitor group (PE model transfected with miR-19a-3p inhibitor), PE + miR19 mimics + SFI group (PE model transfected with miR-19a-3p mimics, and treated with SFI 10 um/ml) and PE + miR19 inhibitor + SFI group (PE model transfected with miR-19a-3p inhibitor, and treated with SFI 10 um/ml). After 24-h transfection, the medium was changed and the cardiomyocytes were incubated with fresh serum-containing medium for 48 h. Then, cells were harvested for immunochemistry, cell surface area analysis, qRT-PCR and protein analysis.

### Protein expression analysis

The myocardial tissues and cardiomyocytes were lysed in NP40 lysis buffer and centrifuged at 12,000 × g at 4 °C for 20 min. The protein concentration was quantitated, and the lysate (20 μg protein) was subjected to SDS-PAGE on 10% polyacrylamide gels and then electrotransferred onto PVDF membranes (Millipore, Billerica, MA, USA). The membranes were then blocked by 5% fat-free milk, and incubated with anti-myocytes specific enhancer factor 2A(MEF2A) (55 kDa; 1:2000), anti-brain natriuretic peptide(BNP) (15 kDa; 1:500), anti-β-myosin heavy chain (β-MHC) (29 kDa; 1:1000), anti- canonical transient receptor protein 1(TRPC1) (91 kDa; 1:10000) and anti-GAPDH (36 kDa; 1:1000) (Abcam, Cambridge, MA, U.S.), at 4 °C overnight. Horseradish peroxidase (HRP)-conjugated secondary antibodies (rabbit or mouse) were used to detect bound antibodies, which were visualized by enhanced chemiluminescence (ECL, Millipore, Billerica, MA, USA) on a VersaDoc 4000 MP (BIO-RAD) workstation. Densitometric analysis employing ImageJ software was used to determine the relative expression level of the target protein, which was then compared with the control (Bethesda, Maryland. USA).

### Statistical analysis

miRNA expression data were compared between control, model and SFI groups by independent Student’s t-test. All data are expressed as mean ± SD and were analyzed by one-way ANOVA. Differences between groups were compared by Fisher’s least significant difference (LSD). All statistical analyses were performed by using SAS 9.0 (SAS Institute, Inc., USA). Two-tailed P < 0.05 showed statistical significance. Statistical comparisons were performed on GraphPad Prism 6.02^28,29^.

### Data availability

The datasets generated during and/or analysed during the current study are not publicly available in that the project is part of the National Natural Science Foundation of China and the subject study has not yet been completed, but they are available from the corresponding author on reasonable request.

## Electronic supplementary material

Supplementary Information
