# Supplementary material for: Shenfu Injection attenuates rat myocardial hypertrophy by up-regulating miR-19a-3p expression
Source: Sci Rep. 2018 Mar 16;8:4660. doi: 10.1038/s41598-018-23137-4 (PMC5856750; doi:10.1038/s41598-018-23137-4)
Supplement: Supplementary file 1 — Supplementary Information [file 41598_2018_23137_MOESM1_ESM.docx]

**Shenfu Injection attenuates rat myocardial hypertrophy by up-regulating miR-19a-3p expression**

Zhu-Jun Mao^1#^, Quan-Long Zhang ^1#^, Jia Shang ^2^, Ting Gao ^2^, Wen-Jun Yuan^2,3*^, Lu-Ping Qin^1*^

^1^Department of Pharmacognosy, Zhejiang Chinese Medical University School of Pharmacy, Hangzhou 310053, ZJ, China

^2^Department of Physiology, Ningxia Medical University, Yinchuan 750004, NX, China

^3^Department of Physiology, Second Military Medical University, Shanghai 200433, China.

^#^ These authors contributed equally to this work

^*^ Corresponding author:

Email address: yuanwj@hotmail.com, Tel.:+86-951-6980173; qinsmmu@126.com, Tel.: +86-571-61768167.

Email address:

Zhu-Jun Mao:maozhujun0107@163.com

Quan-Long Zhang：13484207@qq.com

Jia Shang: 910094513@qq.com

Ting Gao: 1220632648@qq.com


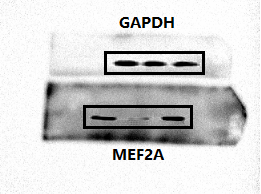


Fig1


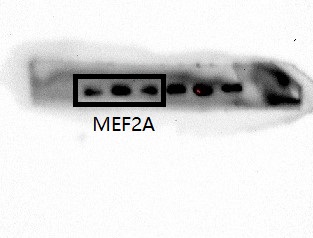


Fig2


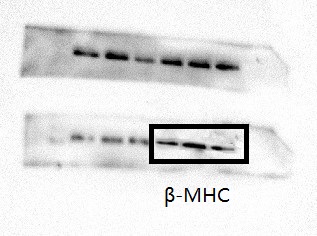


Fig3


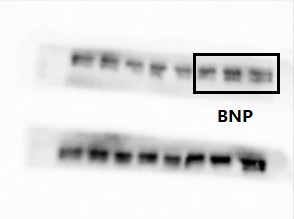


Fig4


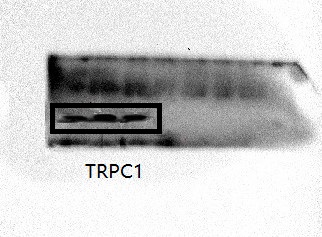


Fig5


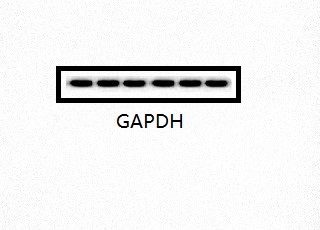


Fig6


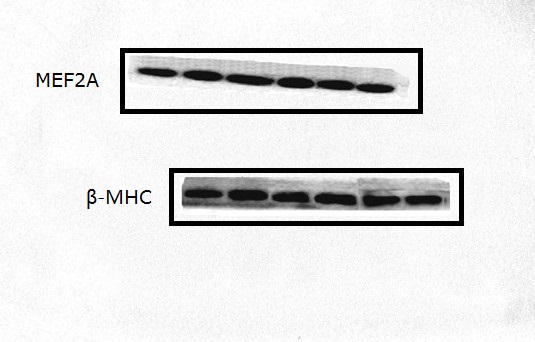


Fig7


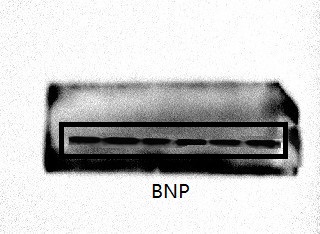


Fig8


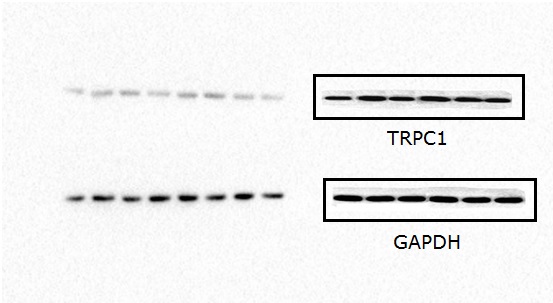


Fig9

**Figure legends:**

Fig 1. The blot of MEF2A protein, from the 293T cells, correspond to the MEF2A protein blot in Fig.3E of the manuscript.

Fig 2. The blot of MEF2A protein, from the rat myocardial tissue, correspond to the MEF2A protein blot in Fig.6A of the manuscript.

Fig 3. The blot of β-MHC protein, from the rat myocardial tissue, correspond to theβ-MHC protein blot in Fig.6A of the manuscript.

Fig 4. The blot of BNP protein , from the rat myocardial tissue, correspond to the BNP protein blot in Fig.6A of the manuscript.

Fig 5. The blot of TRPC1 protein , from the rat myocardial tissue, correspond to the TRPC1 protein blot in Fig.6A of the manuscript.

Fig 6. The blot of GAPDH protein, from the rat myocardial tissue, correspond to the GAPDH protein blot in Fig.6A of the manuscript.

Fig 7. The blots of MEF2A and β-MHC protein, from cardiomyocytes, correspond to the MEF2A and β-MHC protein blots in Fig.6C of the manuscript.

Fig 8. The blot of BNP protein, from cardiomyocytes, correspond to the BNP protein blot in Fig.6C of the manuscript.

Fig 9. The blots of TRPC1 and GAPDH protein ,from cardiomyocytes, correspond to the TRPC1 and GAPDH protein blots in Fig.6C of the manuscript.
